# Tension Pneumomediastinum and Coronary Artery Thrombosis Following a Motorcycle Accident: A Case Report

**DOI:** 10.5811/cpcem.1410

**Published:** 2023-05-27

**Authors:** Samuel G. Rouleau, Martin A.C. Manoukian, Gordon X. Wong, David K. Barnes

**Affiliations:** * University of California Davis Health, Department of Emergency Medicine, Sacramento, California; † University of California Davis Health, Division of Cardiovascular Medicine, Department of Internal Medicine, Sacramento, California

**Keywords:** blunt trauma, coronary artery thrombosis, pneumomediastinum, motorcycle accident, case report

## Abstract

**Introduction**: Tension pneumomediastinum and coronary artery thrombosis (CAT) secondary to blunt polytrauma are, rare yet have the potential for serious complication.

**Case Report**: A 40-year-old man presented to the emergency department following a motorcycle accident. He was found to have multiple orthopedic injuries, pneumothorax, and pneumomediastinum. An electrocardiogram showed myocardial infarction. He developed obstructive shock physiology that resolved with mediastinal percutaneous needle drainage. Subsequent coronary angiography revealed acute thrombosis of the left circumflex artery.

**Conclusion:**

This is a rare case of traumatic tension pneumomediastinum associated with coronary artery thrombosis requiring coronary stenting. Emergency physicians should be mindful of CAT in the setting of blunt chest injury.

## INTRODUCTION

Polytrauma following motorcycle accidents is associated with significant morbidity and mortality. In the United States, motorcycle accidents have a fatality rate 28 times higher than other motor vehicle collisions and represent 14% of all motor vehicle fatalities.^1^ Blunt chest trauma (BCT) is common after motorcycle accidents and can lead to life-threatening injuries including blunt cardiac injury (BCI), pulmonary injury, airway compromise, and damage to the great vessels.

Blunt cardiac injury is associated with approximately one fifth of all deaths caused by BCT in motor vehicle collisions.^2^ Traumatic pneumomediastinum is also associated with increased mortality in BCT.^3^ Although rare, coronary artery injury and coronary artery thrombosis (CAT) are life-threating complications of BCI. Blunt chest trauma causing CAT in the absence of coronary artery dissection has been sparsely reported in the literature.^4^ Herein, we report a unique case of a 40-year-old man who presented with pneumothorax, pneumomediastinum, and CAT following a motorcycle accident.

## CASE REPORT

A 40-year-old man presented to the emergency department (ED) via emergency medical services following a high-speed motorcycle versus automobile collision. Due to respiratory distress and decreased breath sounds in the right lung field, needle decompression was performed on scene by paramedics. Upon arrival to the ED, the patient was hemodynamically stable with a heart rate of 89 beats per minute and blood pressure 120/74 millimeters of mercury (mm Hg). His mentation was intact. Physical exam revealed extensive right-sided chest wall ecchymosis and tenderness.

**Image 1:**
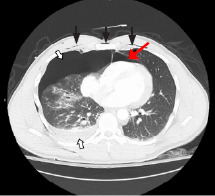
Computed tomography of the chest with intravenous contrast demonstrating subcutaneous emphysema (black arrows), pneumomediastinum (red arrow), and hemopneumothorax (white arrows with black outline).

Point-of-care ultrasound (POCUS) demonstrated reduced lung sliding on the right and no intraperitoneal free fluid; however, cardiac windows were suboptimal and non-diagnostic. Bedside chest radiograph showed multiple right-sided rib fractures with apical and lateral pneumothorax. Computed tomography (CT) demonstrated fractures of right ribs 3–11 with accompanying pneumothorax, sternomanubrial joint dislocation with retrosternal hematoma, and pneumomediastinum (Image 1). Additionally, the patient was found to have a grade 1 liver laceration and first lumbar (L1) vertebral body fracture with 10 mm of retropulsion.

**Image 2:**
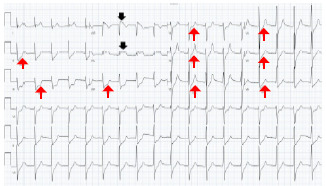
Electrocardiogram with ST-segment elevation in leads aVR and aVL (black arrows), and diffuse ST depression in leads II, III, aVF, and V1–V6 (red arrows).

A 14-French pigtail catheter was placed into the right hemithorax without immediate complication. Twelve-lead electrocardiogram was remarkable for ST-segment elevation in leads aVR and aVL with diffuse ST depression in leads II, III, aVF, and V1–V6 concerning for BCI and prompting cardiology consultation (Image 2). An initial high-sensitivity troponin T returned at 225 nanograms/milliliter (ng/mL) (reference range: <19). During the cardiology team’s evaluation, the patient complained of worsening chest pain and difficulty breathing, becoming hypotensive with a blood pressure of 78/59 mm Hg and tachycardic with a heart rate of 119 bpm.

Repeat POCUS demonstrated bilateral lung sliding and no intra-abdominal free fluid; similarly, the chest tube and pleural drainage apparatus were functioning normally. Again, cardiac windows could not be obtained due to presumed air scattering. Because of significant pneumomediastinum seen on CT, obstructive shock physiology from the mediastinal free air was suspected. An emergent bedside percutaneous needle drainage in the left fifth intercostal space was performed, yielding 9 mL of blood with scant air bubbles. The patient’s blood pressure immediately improved, and cardiac windows were subsequently visualized on POCUS.

**Image 3:**
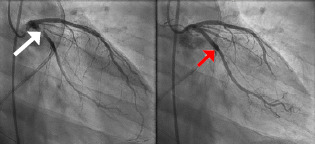
Left: pre-stent right anterior oblique (RAO) view of proximal left circumflex artery sub-total occlusion (white arrow). Right: post-stent RAO view showing recanalized left circumflex artery (red arrow).

With high suspicion for acute myocardial infarction, the patient was taken for emergent coronary angiography. Transthoracic and transesophageal ultrasounds obtained in the cardiac catheterization lab did not demonstrate pericardial fluid or cardiac tamponade. Angiography demonstrated 95% thrombotic occlusion of the proximal left circumflex artery at the origin of the first obtuse marginal branch without evidence of coronary artery dissection (Image 3). Percutaneous coronary intervention (PCI) with aspiration thrombectomy and deployment of two drug-eluting stents resulted in complete restoration of coronary blood flow. The patient was transferred to the surgical intensive care unit.

*CPC-EM Capsule*

What do we already know about this clinical entity?

*Coronary artery thrombosis (CAT) and tension pneumomediastinum are potential causes of morbidity and mortality in blunt chest trauma*.

What makes this presentation of disease reportable?

*A CAT in the absence of coronary dissection is rare and may be attributable to the vascular stasis caused by obstructive shock physiology*.

What is the major learning point?

*Clinicians should consider CAT in polytrauma patients with chest pain and concerning ECG findings, particularly when tension physiology occurs*.

How might this improve emergency medicine practice?

*Early involvement of multidisciplinary specialists and careful consideration of interventional strategies can improve patient outcomes in CAT associated with polytrauma*.

Aspirin and ticagrelor were initiated immediately after PCI. Norepinephrine and amiodarone were administered for cardiogenic shock and non-sustained ventricular tachycardia, respectively, both of which resolved within 48 hours. Following cardiac stabilization, the inpatient team addressed the patient’s spinal injury, although urgent operative intervention was deferred because of hemodynamic instability and the bleeding risk associated with antiplatelet therapy. Prior to spinal fixation on hospital day three, ticagrelor was changed to tirofiban infusion, which was held prior to the first incision and resumed postoperatively. Ticagrelor was re-started following the removal of a spinal drain on postoperative day two. The patient remained stable for the remainder of hospitalization and was discharged on aspirin and ticagrelor on hospital day 14.

On a follow-up phone call with the patient almost six months after the injury, he reported that he was back to working full time and feeling fully recovered. He recalled the accident and arriving to the ED, although he did not recall undergoing tube thoracostomy placement or percutaneous needle drainage. He was advised to continue aspirin and ticagrelor for at least six months.

## DISCUSSION

This patient’s ED course and management were complex due to the number and severity of his injuries. He developed obstructive shock physiology. We hypothesize that both blood and air in the mediastinum resulted in obstructive shock. In general, the differential diagnoses of obstructive shock in the setting of BCT include tension pneumothorax, pneumomediastinum, pneumopericardium, hemopericardium, and large retrosternal hematoma.^5^–^9^ The presence of retrosternal hematoma on CT and aspiration of blood during percutaneous needle drainage suggest that the retrosternal hematoma may have contributed to the obstructive shock physiology by exerting extrapericardial force on the heart. Retrosternal hematomas have been reported to accumulate over time and result in delayed decompensation hours after injury.^10^ Although the rate of fluid accumulation is typically understood to be a more important factor in causing obstructive shock physiology and has been well defined for cardiac tamponade, the volume and rate of accumulation necessary for a retrosternal hematoma to cause obstructive shock secondary to extra-pericardial compression is unknown.

In addition to the retrosternal hematoma, pneumomediastinum identified on CT and the presence of air bubbles during percutaneous needle drainage suggest that the air in the mediastinum also contributed to the development of obstructive shock. Traumatic pneumomediastinum in BCT can be caused by several injuries, such as tracheoesophageal injury, alveolar injury, hollow viscus injury, or facial (sinus) injury.^5^ In this case, we suspect pneumomediastinum was likely secondary to extensive right-sided pneumothorax, pulmonary injury, and alveolar injury combined with the Macklin effect. The Macklin effect occurs when blunt trauma ruptures alveoli allowing air to dissect along bronchovascular sheaths resulting in mediastinal air.^5^ Although only a small amount of air was aspirated, needle insertion may have created a communication between the mediastinum and right hemithorax where a functional thoracostomy tube was already in place, thereby allowing for additional drainage of air. While the exact etiology of the obstructive shock physiology remains unknown, we hypothesize that the presence of blood (retrosternal hematoma) and air (pneumomediastinum) played significant roles. The bedside percutaneous needle drainage resolved the hemodynamic instability and allowed patient care to proceed.

Isolated CAT following BCT in the absence of concurrent coronary artery dissection is a rare complication that has been reported sparingly in the literature; therefore, its epidemiology is difficult to define.^4^ As with coronary artery dissection, there is predilection for the left anterior descending artery, which is hypothesized to be due to its anatomically vulnerable location immediately behind the sternum.^11^ The pathophysiology of thrombus formation is hypothesized to involve endothelial injury to the coronary artery wall caused by direct blunt trauma as well as indirect kinetic energy transmission that causes further damage or stasis of flow.^4^

In this instance, the presence of a CAT in the left circumflex artery may suggest an etiology other than direct trauma. It has been postulated that tension physiology from a pneumothorax can lead to reduced coronary artery blood flow thereby causing vascular stasis and thrombus formation.^12^ Extracardiac forces from pneumomediastinum and retrosternal hematoma may have compressed the left circumflex artery, leading to vascular congestion and thrombosis. The initial high-sensitivity troponin, drawn prior to the development of obstructive shock physiology, was 225 ng/mL and consistent with BCI. However, after development of obstructive shock and subsequent percutaneous needle drainage of retrosternal hematoma and pneumomediastinum, troponin increased to 1,161 ng/mL, eventually peaking at 4,012 ng/mL. While a delayed rise in troponin is expected after BCI, the significant rise in troponin after the development of obstructive shock support our hypothesis. Importantly, this patient had no risk factors for coronary artery disease, which also support our hypothesis.

Co-occurrence of an unstable spinal injury and liver injury complicated the decision to administer anti-platelet and anti-coagulant medication in this case. Antiplatelet and anticoagulation therapy to treat CAT must be weighed against the risk of hemorrhage in a critically injured trauma patient. Severity of concomitant injuries, compressibility of current or possible hemorrhage, underlying hematologic conditions, and current medication usage must all be considered. Contrary to aspirin, clopidogrel, and ticagrelor, whose antiplatelet effects may continue for days, heparin and tirofiban are favored in patients with CAT. Both agents are associated with low to medium risk of bleeding because of their relatively short half-life and titratability.^13^ While tranexamic acid administration has been shown to decrease mortality in multisystem trauma, it is also associated with an increased risk of thrombotic events and should be avoided in patients with known or suspected thrombosis.^14^ This patient received aspirin and ticagrelor after stent placement but was subsequently switched to tirofiban to allow titration. After surgical fixation of the L1 fracture on hospital day three and removal of spinal drain on hospital day five, ticagrelor was restarted, and the patient was discharged on dual antiplatelet therapy without any major bleeding events.

## CONCLUSION

Coronary artery thrombosis and pneumomediastinum are uncommon complications of blunt cardiac injury, yet both injuries are associated with significant complications and present diagnostic challenges and treatment dilemmas, particularly in the setting of multisystem trauma. Emergency clinicians must be cognizant of the complications of blunt chest trauma and be prepared to identify and stabilize these patients while definitive care is pending.
